# Distribution of the *pco* Gene Cluster and Associated Genetic Determinants among Swine *Escherichia coli* from a Controlled Feeding Trial

**DOI:** 10.3390/genes9100504

**Published:** 2018-10-18

**Authors:** Gabhan Chalmers, Kelly M. Rozas, Raghavendra G. Amachawadi, Harvey Morgan Scott, Keri N. Norman, Tiruvoor G. Nagaraja, Mike D. Tokach, Patrick Boerlin

**Affiliations:** 1 Department of Pathobiology, Ontario Veterinary College, University of Guelph, 50 Stone Rd. E., Guelph, ON N1G 2W1, Canada; gchalmer@uoguelph.ca; 2 Department of Veterinary Pathobiology, College of Veterinary Medicine and Biomedical Sciences, Texas A&M University, College Station, TX 77843, USA; kmrozas@cvm.tamu.edu (K.M.R.); hmscott@cvm.tamu.edu (H.M.S.); 3 Department of Clinical Sciences, College of Veterinary Medicine, Kansas State University, Manhattan, KS 66506, USA; agraghav@vet.k-state.edu; 4 Department of Veterinary Integrative Biosciences, College of Veterinary Medicine and Biomedical Sciences, Texas A&M University, College Station, TX 77843, USA; knorman@cvm.tamu.edu; 5 Department of Diagnostic Medicine/Pathobiology, College of Veterinary Medicine, Kansas State University, Manhattan, KS 66506, USA; tnagaraj@vet.k-state.edu; 6 Department of Animal Sciences & Industry, College of Agriculture, Kansas State University, Manhattan, KS 66506, USA; mtokach@k-state.edu

**Keywords:** copper, resistance, swine, *Escherichia coli*

## Abstract

Copper is used as an alternative to antibiotics for growth promotion and disease prevention. However, bacteria developed tolerance mechanisms for elevated copper concentrations, including those encoded by the *pco* operon in Gram-negative bacteria. Using cohorts of weaned piglets, this study showed that the supplementation of feed with copper concentrations as used in the field did not result in a significant short-term increase in the proportion of *pco*-positive fecal *Escherichia coli*. The *pco* and *sil* (silver resistance) operons were found concurrently in all screened isolates, and whole-genome sequencing showed that they were distributed among a diversity of unrelated *E. coli* strains. The presence of *pco*/*sil* in *E. coli* was not associated with elevated copper minimal inhibitory concentrations (MICs) under a variety of conditions. As found in previous studies, the *pco*/*sil* operons were part of a Tn*7*-like structure found both on the chromosome or on plasmids in the *E. coli* strains investigated. Transfer of a *pco*/*sil* IncHI2 plasmid from *E. coli* to *Salmonella* *enterica* resulted in elevated copper MICs in the latter. *Escherichia coli* may represent a reservoir of *pco*/*sil* genes transferable to other organisms such as *S. enterica*, for which it may represent an advantage in the presence of copper. This, in turn, has the potential for co-selection of resistance to antibiotics.

## 1. Introduction

As restrictions on the use of antimicrobial agents for the purpose of growth promotion and disease prevention in farm animals are increasing, alternatives to these agents are becoming more popular. Feed supplementation with copper is one of the most frequently used, particularly in the swine industry [1]. The copper concentrations used in swine feed for growth promotion are relatively high and usually in the range of 100 to 250 ppm [2].

Bacteria developed mechanisms to cope with high concentrations of copper. In Gram-positive bacteria, the most well-known mechanism is the *tcrB* gene [3,4], which provides a selective advantage to intestinal enterococci in swine and cattle [5,6,7]. It also seems to be involved in the co-selection of bacteria resistant to antimicrobial agents of importance for both veterinary and human medicine [6,8]. Several tolerance and homeostasis mechanisms were described in Gram-negative bacteria and in *Enterobacteriaceae* in particular (for a review, see, for instance, References [9,10,11]). Although most are chromosomally encoded and present in the majority of bacteria from the species in which they reside, one of them initially found in *Escherichia coli* was shown to be plasmid-borne and not present in every isolate of the species [12]. The *pco* gene cluster associated with this system was later characterized in more detail [13] and shown to consist of seven genes (*pcoA*, *B*, *C*, *D*, *R*, *S*, and *E* [14,15,16]). This cluster was found in a variety of *Enterobacteriaceae* species and, depending on bacterial species and strain, the associated copper tolerance phenotype was variable, both in terms of copper minimal inhibitory concentration and inducibility [13]. Since then, several studies showed that *pco* genes are not always plasmid-borne but can also regularly be found on the chromosome of *Enterobacteriaceae* species, including *Salmonella enterica* and *E. coli* [17,18,19]. This spread and mobility may be related to the location of the *pco* genes on a Tn*7*-like transposon [17]. This Tn*7*-like element frequently carries both the *pco* gene cluster and the *sil* gene cluster [17] associated with silver tolerance [20]. Investigations on silver and copper tolerance in *S. enterica* isolates from Portugal showed a clear association between the presence of *sil* genes and copper tolerance, while the presence of *pco* genes did not seem to show any evident correlation with this phenotype [18,21]. Similarly, recent experimental studies on the effect of feed supplementation with copper on fecal *E. coli* and on the fecal metagenome of swine did not demonstrate any clear or systematic selective effect for *pco* genes [2,22]. These results suggest that either the concentrations of copper used in feed (125 ppm) may have been too low to have such an effect, or the presence/absence of the *pco* genes did not affect the tolerance of *E. coli* and other bacteria to elevated copper concentrations under the conditions found in the gut of the animals. However, a negative association was observed between copper supplementation and resistance to antimicrobials, as well as resistance to extended-spectrum cephalosporins in particular [2]. Also, an association between *pco* genes and the *tet*(B) tetracycline resistance gene was detected in *E. coli*, while these two genes were negatively associated with the *bla*_CMY_ and *tet*(A) genes encoding for extended-spectrum cephalosporins and tetracycline resistance, respectively [2].

Based on these observations, the objectives of this study were (a) to replicate the previous experiments of Agga and collaborators [2] and reassess the associations between the *pco* genes and *tet*(A), *tet*(B), *bla*_CMY_, and *bla*_CTX-M_ among *E. coli* from groups of swine subjected to diverse combinations of copper and tetracycline feed supplementation; (b) to use whole-genome sequencing to assess the genetic diversity and clonal relationships of *E. coli* isolates recovered from these experiments and carrying diverse combinations of these genes; (c) to compare the copper susceptibility and genome sequences of selected isolates with plasmid-borne and chromosomally encoded *pco* genes; and (d) to transfer *E. coli* plasmids carrying the *pco* and *sil* gene clusters into *S. enterica* by conjugation, and assess the associated copper susceptibility. These objectives related to the use of copper in feed and its effect on copper tolerance in *E. coli* were part of a broader study on alternatives to antibiotics [23]. The latter also included the use of zinc and oregano oil, but is not discussed here.

## 2. Materials and Methods

### 2.1. Experiment Design

The Kansas State University Institutional Animal Care and Use Committee approved the protocol for this experiment (AUP # 3135). The study was conducted at the university’s Segregated Early Weaning Facility in Manhattan, KS. Each pen (1.22 × 1.22 m) had metal tri-bar flooring, one four-hole self-feeder, and a cup waterer to provide ad libitum access to feed and water. This experiment was also described in a publication by Feldpausch and collaborators [23].

A total of 350 piglets (21 days old) were assigned to one of 70 pens (five piglets per pen), which were then randomly assigned to each of the 10 in-feed treatments arranged in a 2 × 2 × 2 (+2) factorial design. In detail, the ten dietary treatments were (1) a basal swine diet fully meeting National Research Council (NRC) nutritional guidelines, including 16.5 ppm of supplemental copper and 165 ppm of supplemental zinc (control group); (2) a basal diet supplemented with 125 ppm of copper provided by copper sulfate; (3) a basal diet supplemented with zinc at 3000 ppm of zinc provided by zinc oxide; (4) a basal diet supplemented with oregano premix containing 5% oregano oil (Regano 500; Ralco-mix Products, Marshall, MN, USA); (5) a basal diet with both 125 ppm of copper and zinc at 3000 ppm; (6) a basal diet with both 125 ppm of copper and oregano premix; (7) a basal diet with both zinc at 3000 ppm and oregano premix; (8) a basal diet containing copper, zinc, and oregano premix; (9) a basal diet containing a preventive level of chlortetracycline (CTC) (22 mg/kg body weight (BW); High CTC); and (10) a basal diet containing a subtherapeutic level of CTC (4 mg/kg BW; Low CTC). These latter treatment groups (9 and 10) did not interact with other main treatment factors (Zn, Cu, and oregano oil) in the study design, so as to assess the impact of antimicrobial alternatives versus both true negative controls and the “existing standard controls” represented by antimicrobial use groups. The basal diet consisted of corn, soybean meal, vitamins, amino acids, and trace mineral supplements per NRC requirements.

The study lasted 49 days with an initial seven days of acclimation, and 28 days of feeding trial, followed by 14 days of washout phase. Three fresh fecal samples were collected from random pigs in each pen by gentle rectal massage at days 0 and 28 of the feeding trial. Fecal samples were transported to the laboratory for further processing. The fecal samples were thoroughly mixed with 50% glycerol (1:1) and stored at −80 °C. Laboratory personnel were blinded to the treatment groups.

### 2.2. Selection of Isolates and Detection of *pco*

A total of 420 samples, 210 from day 0 (for pre-treatment effect) and 210 from day 28 (maximum treatment effect), were subjected to bacteriological culture and quantified for *E. coli* using standard isolation techniques and spiral plating. Briefly, one gram of 50:50 glycerol and feces were diluted in 9 mL of phosphate-buffered saline (PBS). A 50-μL aliquot of the fecal suspension was spiral-plated onto each of MacConkey agar, MacConkey agar supplemented with 16 mg/L tetracycline, and MacConkey agar supplemented with 4 mg/L ceftriaxone using an Eddy Jet 2 spiral plater (Neu-tec Group Inc., Farmingdale, NY, USA). Crude quantification values were determined by the Flash & Go Automatic Colony Counter (Neu-tec Group Inc.). A single, randomly selected colony was used from a plain MacConkey plate and confirmed as *E. coli* by lactose fermentation and an indole test; the species identity was also later confirmed with Illumina-based DNA sequencing. Isolates were preserved at −80 °C in protectant CryoBeads™ for further characterization.

Antimicrobial susceptibility testing was conducted by broth microdilution using the Sensititre™ system (TREK, Thermo Scientific Microbiology, Oakwood Village, OH, USA) and Sensititre™ NARMS Gram-negative plates (CMV3AGNF) on 403 *E. coli* isolates. *Escherichia coli* ATCC 25922, *Escherichia coli* ATCC 35218, *Pseudomonas aeruginosa* ATCC 27853, *Staphylococcus aureus* ATCC 29213, and *Enterococcus faecalis* ATCC 29212 were used as quality control strains. Plates were incubated at 37 °C for 18 h and read on a Sensititre OptiRead™ (TREK). The results were interpreted according to Clinical and Laboratory Standards Institute (CLSI) guidelines [24]. Intermediate isolates were interpreted as susceptible for binary statistical analyses.

Detection of *pco*, tetracycline, and extended-spectrum cephalosporin resistance genes was performed by PCR with the primers described in Table 1. Thermocycling conditions were the same as those defined in the respective references. Amplicons were visualized by horizontal gel electrophoresis and ultraviolet (UV) imaging.

### 2.3. Copper Susceptibility

Susceptibility to copper was analyzed by broth microdilution for four randomly selected *pco*/*sil*-positive *E. coli*, and two *pco*/*sil*-negative isolates. Isolates were from day 0 (KSC9, 27, 64, and 207) and day 28 (KSC857 and 1031), from animals within the copper treatment group (KSC27, 857, and 1031) and from those without (KSC9, 64, and 207). A stock solution of 400 mM copper(II) sulfate (Sigma-Aldrich, St. Louis, MO, USA) was prepared in double-distilled water (ddH_2_O), and filter-sterilized. A non-serial dilution range (0, 4, 8, 16, 20, 24, 36, 48, 64, and 100 mM) was prepared in Mueller–Hinton II broth, cation-adjusted (Becton Dickinson, Franklin Lakes, NJ, USA), and each dilution was adjusted to pH 7.2 using 5 M NaOH [11]. Bacterial suspensions of a 0.5 McFarland standard were diluted 1/100, and 50 μL of this suspension was inoculated in a 96-well plate with 50 μL of the copper dilutions, resulting in halving of the initial copper concentrations. Microplates were incubated at 37 °C for 16 h, under both aerobic and anaerobic conditions. Minimum inhibitory concentration (MIC) was defined as the first concentration without visible growth. Minimum bactericidal concentrations (MBCs) were determined by removing 10 μL from wells that showed no visible growth, and plating them on Mueller–Hinton II agar plates for incubation at 37 °C for 16 h. The ATCC 25922 *E. coli* strain (*pco*/*sil*-negative) was used as a negative control for susceptibility testing.

Minimum inhibitory concentrations were also determined using agar plate dilutions of copper, as described by Mourão and collaborators [21]. Briefly, copper dilutions of 0, 0.5, 1, 2, 4, 8, 12, 16, 20, 24, 28, 32, and 36 mM were prepared in Mueller–Hinton II agar, and the pH was adjusted as above. One microliter of an approximate 10^7^ colony forming units (CFU)/mL culture was pipetted onto the surface of each plate. Growth at 37 °C in both aerobic and anaerobic conditions was assessed after 16 h.

### 2.4. Expression of *pco* by Complementary DNA Synthesis and Real-Time PCR

Three *E. coli* isolates were selected randomly (two *pco*/*sil*-positive and one negative control, none of which were isolated from copper-treated animals) for determining the expression of *pco* under aerobic conditions, with and without induction with low concentrations of copper, performed as previously described [15,27]. Briefly, isolates were plated overnight at 37 °C on Luria–Bertani (LB) agar (Becton Dickinson) plates. A single loop of bacteria was inoculated into 1 mL of LB broth, and vortexed; 200 μL of this suspension was inoculated into 20 mL of LB broth supplemented with 0, 1 mM, and 5 mM copper(II) sulfate and incubated at 37 °C for approximately 2.5 h, until optical densities of 0.5 were reached at 600 nm. Broth microdilution MICs were performed again as above under aerobic conditions to observe any effect of this induction on copper tolerance. In parallel, 10 mL of broth was centrifuged, and the resulting pellet was resuspended in 1 mL of RNAlater (QIAGEN Inc., Valencia, CA, USA). Total RNA was extracted using an RNAeasy Mini kit (QIAGEN), according to the manufacturer’s instructions. An additional DNase step was performed to ensure all traces of DNA were removed, and was verified by a *pco* PCR using 1 μL as a template. RNA was quantified using a BioAnalyzer 2100 instrument (Agilent Technologies, Santa Clara, CA, USA), and 100 ng of each RNA preparation was used for complementary DNA (cDNA) synthesis using an Applied Biosystems High-Capacity cDNA Reverse Transcription kit (Thermo Fisher Scientific, Carlsbad, CA, USA).

PCR was performed to amplify gene fragments to be used for cloning into a plasmid vector, for use as a standard curve for real-time PCR. Amplicons of the *pcoA* and *pcoD* genes were produced using primers forward *pcoA* (pcoA_F), CGGGTATGCAAAGTCATCCT; reverse *pcoA* (pcoA_R), TTGATCAGCGTGATCCTGAG; and pcoD_F, AAGCGGTGTCAGACATGAAA; pcoD_R, GATGGGTCAGATCGCTCAGT, respectively. As controls, two housekeeping gene amplicons for *hcaT* (HcaT major facilitator superfamily transporter) and *rrsA* (16S ribosomal RNA) were amplified using primers hcaT_F, CTGATGCTGGTGATGATTGG; hcaT_R, CAATGCAGAATTTGCACCAC; and rrsA_F, CGGACGGGTGAGTAATGTCT; rrsA_R, GTTAGCCGGTGCTTCTTCTG, respectively. Each amplicon was cloned into a pCR 2.1-TOPO plasmid vector, using an Invitrogen TOPO-TA cloning kit (Thermo Fisher Scientific). Inserted sequences were confirmed by DNA sequencing, and plasmid DNA was prepared using a Plasmid Midi Kit (QIAGEN). Plasmid DNA was quantified using Quant-IT Picogreen dsDNA reagent (Thermo Fisher Scientific) and read using a DTX 880 Multimode detector (Beckman Coulter, Brea, CA, USA). Gene copy numbers were then predicted by the DNA concentration divided by the molecular weight of the plasmid.

Real-time PCR was used to quantify the expression of each *pcoA*, *pcoD*, *hcaT*, and *rrsA* gene using primers internal to the fragments described above. In triplicate, 1 μL of cDNA or plasmid standards were added to 19 μL of LightCycler 480 SYBR Green I Master (Roche Diagnostics, Indianapolis, IN, USA) containing 250 nM of each primer. Primers used for the quantification of *pco* expression were RT_pcoAF, TGGTTGATATGCAGGCGATG; RT_pcoAR, TCCGCGTACGTGAGAACCTT; and RT_pcoDF, GTCAGGCTCTGTGCCCTGTT; RT_pcoDR, CCCACTCATCGTCATCAGCA. Housekeeping gene primers used for *hcaT* and *rrsA* were those described by Zhou and collaborators [28].

### 2.5. Next-Generation Sequencing

A subset of 82 isolates was selected to represent suspected extended-spectrum β-lactamase (ESBL)-producing isolates and isolates with elevated ciprofloxacin MICs (based on Sensititre phenotypes and ciprofloxacin MICs of ≥0.05 mg/L; *n* = 26), isolates carrying the *bla*_CMY_ gene (*n* = 26), and a representative sample of isolates with resistance phenotypes determined by Sensititre (*n* = 30). In addition, all 34 *E. coli* carrying the *pcoD* gene were also included. Genomic DNA was prepared for MiSeq sequencing (Illumina, San Diego, CA, USA) for all of these 116 isolates using a QIAamp DNA extraction kit (QIAGEN), and libraries were prepared using a Nextera XT kit (Illumina). Achtman sequence types were determined with the SRST2 plugin for BaseSpace Labs (Illumina) using the MiSeq paired-end reads, where sufficient read quality was obtained. These reads were also used for core-genome multilocus sequence typing (cgMLST) (EnteroBase typing scheme) using the wgMLST application for BioNumerics v7.6 (Sint-Martens-Latem, Belgium). Single-nucleotide polymorphism (SNP) analysis of each *pco* gene cluster (*pcoEABCDRSE*) was also performed using the wgSNP analysis tool from BioNumerics.

DNA was also prepared for four of the isolates tested for susceptibility to copper and harboring *pco* (KSC9, KSC64, KSC207, and KSC1031) using a MasterPure DNA Purification Kit (Epicentre, Madison, WI, USA) for PacBio RS II sequencing (Pacific Biosciences, Menlo Park, CA, USA). Sequencing and assembly of these four isolate genomes were performed at the McGill University and Génome Québec Innovation Centre, Montreal, QC, Canada. PacBio sequencing assembly was completed on chromosome and plasmid assemblies of sheared large inserts (~20 kbp) using the de novo genome assembly pipeline Hierarchical Genome Assembly Process (HGAP); alignments were further polished using the Quiver consensus algorithm. Genomes and/or plasmids (pMRGN207 and pMRGN1031) containing the *pco* gene cluster were uploaded to GenBank under BioProject PRJNA355857. Annotations were performed using the NCBI Prokaryotic Genome Annotation Pipeline (PGAP) version 4.0.

### 2.6. Conjugation of a *pco*/*sil* Plasmid into Salmonella

Based on the known plasmid-borne location in two of our sequenced *E. coli* isolates, we attempted to transfer these plasmids into *Salmonella* recipients to observe any change to copper susceptibility. Six *Salmonella* isolates were selected at random from a large collection of isolates maintained by the Canadian Integrated Program for Antimicrobial Resistance Surveillance (CIPARS) between 2008 and 2011. Isolates were screened for *pco* and *sil* by PCR, and three *pco*/*sil*-positive and three *pco*/*sil*-negative were used for copper susceptibility testing. *Escherichia coli* isolates KSC207 and KSC1031 were used as plasmid donor strains, with *pco*/*sil* plasmid incompatibility types HI2 and FII, respectively (as determined by DNA sequence analysis), while *pco*/*sil*-negative *Salmonella* isolates SA8197 (serovar Kentucky) and SA82540 (serovar Infantis) were used as recipients. Briefly, 50 µL of donor and 100 µL of recipient overnight broth cultures were mixed, plated on LB agar, and incubated overnight to facilitate conjugation. Growth was then resuspended in LB broth, and plated on Brilliant Green agar to inhibit the growth of donor *E. coli*, as both plasmids carried a tetracycline resistance determinant—*tet*(A) and *tet*(B), respectively. Additionally, 12 µg/mL tetracycline was included in the selective plates. Up to five pink-colored colonies were then sub-cultured for purity, and indole testing and PCR for *pco* and *sil* were used to confirm transfer. These transconjugant *Salmonella* were then used for copper susceptibility testing as before using anaerobic agar dilution, and performed in triplicate.

### 2.7. Statistics

Descriptive and inferential statistical methods were performed using Stata version 15 (StataCorp, College Station, TX, USA). Categorical data (i.e., resistance phenotypes and presence of genes) were tabulated and cross-tabulated to explore bivariable associations between treatments and the following outcomes: (1) presence of phenotypic resistance to (a) tetracyclines, (b) cefoxitin, for preliminary classification of ESBL (susceptible) versus AmpC (resistant) producing β-lactamases, (c) third-generation cephalosporins, and (d) multidrug resistance (MDR) count (integer count), out of 14 antimicrobials tested on a broth microdilution panel; and (2) presence of resistance genes for (a) tetracyclines (*tet*(A) and *tet*(B)), (b) extended-spectrum cephalosporins (*bla*_CMY-2_ and *bla*_CTX-M_), and (c) metal resistance genes (*pco* and *sil*). Likelihood ratio χ^2^ or Fisher’s exact tests (when zero-cells were abundant) were used in bivariable analyses; statistical significance was determined at *p* < 0.05. Differences in growth (log_10_ CFU) of coliforms on plain versus antimicrobial (tetracycline or ceftriaxone) impregnated plates were examined by unpaired *t*-tests. Multivariable mixed logistic (binary outcomes) and linear models (count and log_10_ CFU outcomes) using a four-way factorial design (plus two additional indicator variables for low- and high-dose CTC) were built and assessed for each of the binary response (logistic) and quantitative (log_10_ CFU differences) endpoints. Full factorial models were subjected to reduction, firstly removing non-significant interaction terms and then main effects. Of note, in-feed copper (low versus high), sampling day (0 versus 28), and their interaction were always retained marginal means estimated with *p*-values representing the post hoc multiple comparisons adjusted using Bonferroni’s correction.

## 3. Results

### 3.1. Resistance Determinants

Not all samples (*n* = 420) yielded lactose- and indole-positive isolates. A total of 403 *E. coli* isolates were included in this analysis. Thirty-four of the 403 isolates (8.4%) were positive for *pco* by PCR. Twelve isolates carried a *bla*_CTX-M_ gene (3.0%), and 60 carried *bla*_CMY_ (14.9%, all variant CMY-2). All but two isolates (99.5%) were positive for *tet*(A) or *tet*(B) (121 (30.0%) and 267 (66.3%), respectively); 13 isolates (3.2%) carried both of these tetracycline resistance genes.

All *bla*_CTX-M_ sequences encoded the CTX-M-27 variant, and all *bla*_CTX-M_-positive isolates were *pco*-negative, but *tet*(B)-positive; 11 of these 12 isolates were ST744 and had an ampicillin/ceftriaxone/ciprofloxacin/nalidixic acid/tetracycline resistance phenotype.

### 3.2. Associations of Copper Treatment Groups with *pco* Prevalence

Multi-level model-adjusted estimates of the occurrence of the *pco* gene were initially unstable in the presence of the full factorial specification. A reduced model containing copper (forced into model), day (and its interaction), and low- versus high-dose CTC yielded a model significant at *p* < 0.03. Copper did not select for the *pco* gene (*p* = 0.249); that is, copper supplemented at NRC requirements yielded 0.07 (95% confidence intervals (CIs): 0.04–0.11) of isolates with the gene, versus 0.11 (95% CIs: 0.04–0.17) in the group supplemented with copper beyond nutrient needs. Likewise, copper did not appear to select for any of the additional microbiological endpoints (*p* > 0.05; data not shown). Low-dose CTC did select (*p* = 0.001) for increased *pco* with 0.28 (95% CIs: 0.11–0.46) of the isolates in the low-dose group harboring the gene versus 0.06 (95% CIs: 0.04–0.09) in pigs not receiving any CTC. The difference between the high-dose CTC and each of the other two levels was not significant (0.12; 95% CIs: 0.00–0.24). Of note, though unexplained by the field trial study design, the *pco* gene was significantly (*p* = 0.012) associated with a lower MDR count; however, most of this difference was due to a lack of the highest MDR counts (maximum with *pco* present = 7 versus 11 in *pco*-negative isolates). No associations were significant (*p* > 0.05) among the genes tested, with the notable exception of *pco*/*sil* for which there was complete agreement (34/34; Fisher’s exact test, *p* < 0.0001).

### 3.3. Susceptibility to Copper and Expression of *pco*

Susceptibility to copper by broth microdilution ranged from 12 to 18 mM in all seven *E. coli* isolates tested (Table 2). The presence of the *pco* gene cluster did not appear to have an effect on susceptibility to copper under both aerobic and anaerobic conditions, nor did the use of broth or solid media. All MBC values were identical to their respective MIC results. The ATCC 25922 (*pco*/*sil*-negative) isolate had an MIC and MBC of 12 mM. For the *Salmonella* isolates, no systematic differences were observed when using broth dilution under anaerobic conditions (MICs of 12 to 18 mM), but the MICs of the *pco*/*sil*-positive and -negative isolates differed when tested on agar, with values of 24 mM and 4 mM, respectively (Table 2).

Induction with 1 mM and 5 mM under aerobic conditions appeared to have no effect on the MIC of each isolate tested (data not shown). RNA extraction and cDNA analysis also showed no significant change in *pcoA* or *pcoD* transcription, observed by real-time PCR. Using *hcaT* as the reference gene (expression of *rrsA* expression was considerably higher than all other genes, and was not used in the analysis), expressions of *pcoA* and *pcoD* were measured as the “target” in all samples using relative quantification. The average adjusted crossing point (CP) across all three induction concentrations was 31.9 (±1.7) for *pcoA*, and 32.1 (±1.9) for *pcoD*.

### 3.4. Next-Generation Sequencing and cgMLST

**Figure 1:**
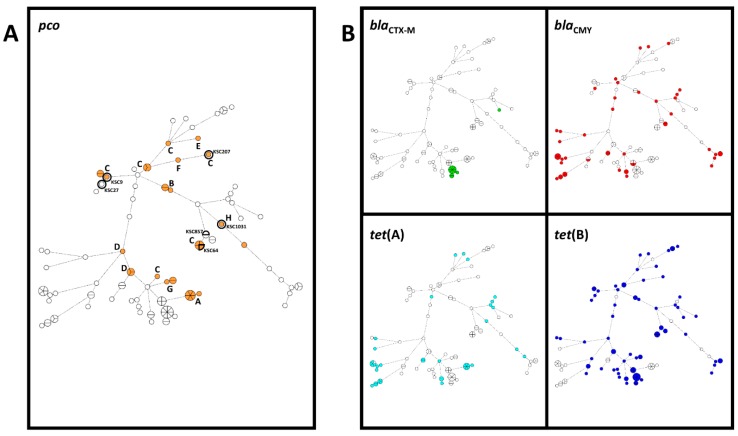
Minimum spanning tree of 116 *Escherichia coli* isolates, using core-genome multilocus sequence typing (MLST) analysis comprising 2513 genes (BioNumerics *E. coli*/*Shigella* EnteroBase scheme). A tree with the highest resampling support is shown, using 1000-resampling bootstrapping. (**A**) Isolates carrying the *pco* gene cluster are highlighted in orange; (**B**) isolates carrying the resistance genes *bla*_CTX-M_, *bla*_CMY_, *tet*(A), and *tet*(B) are indicated. Letters in 1A indicated single-nucleotide polymorphism (SNP) types found in Figure 2. Circles containing multiple sections indicate multiple isolates within a core-genome sequence type. Isolates used for minimum inhibitory concentration (MIC) testing are also highlighted and labeled in 1A.

**Figure 2:**
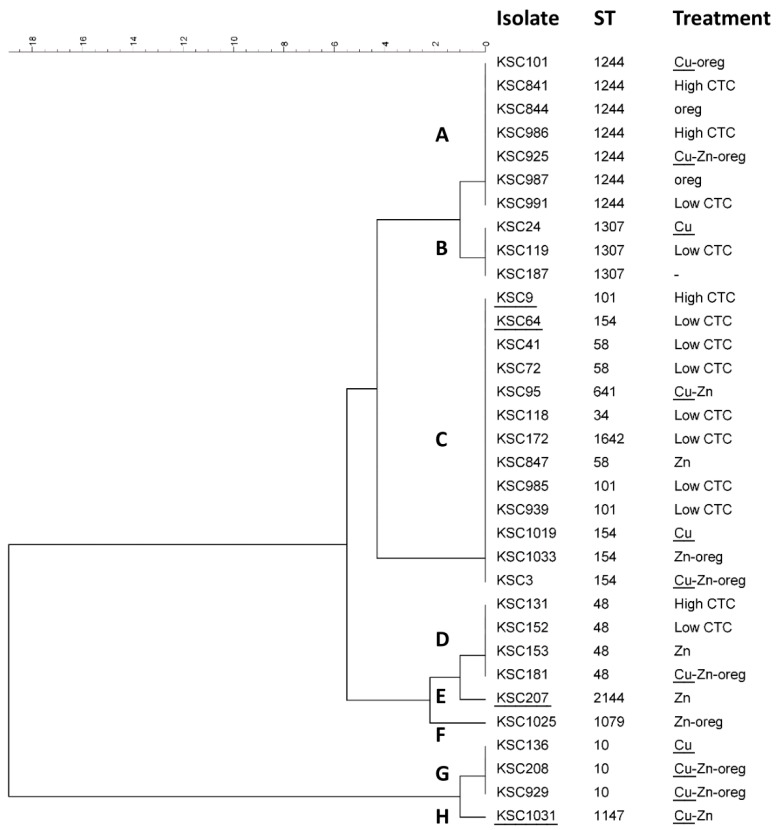
Phylogenetic analysis of the *pcoEABCDRSE* gene cluster (5487 bp) of all *pco*-positive isolates in this study, using a categorical (differences) similarity coefficient and unweighted pair group with arithmetic mean (UPGMA) cluster analysis. Treatment groups, including Cu (copper), Zn (zinc), oreg (oregano oil), and high/low-dose chlortetracycline (CTC) are shown. Letters indicating identical SNP groups are also shown in Figure 1A. Isolates used for MIC testing are underlined. ST: sequence type.

All 116 *E. coli* isolates selected for MiSeq sequencing were analyzed using the BioNumerics software (Figure 1). All 34 *pco*-positive isolates recovered during this study also carried the *sil* gene cluster (determined by reference mapping of MiSeq reads), and none of the 82 *pco*-negative isolates carried any *sil* gene. Core-genome MLST analysis of the MiSeq data showed a random distribution of *pco*/*sil* among sequence types and no association with a specific clonal lineage (Figure 1). Further *pco* SNP analysis showed the gene cluster to be highly conserved within a sequence type (ST), but had some variation between most STs (Figure 2).

**Figure 3:**
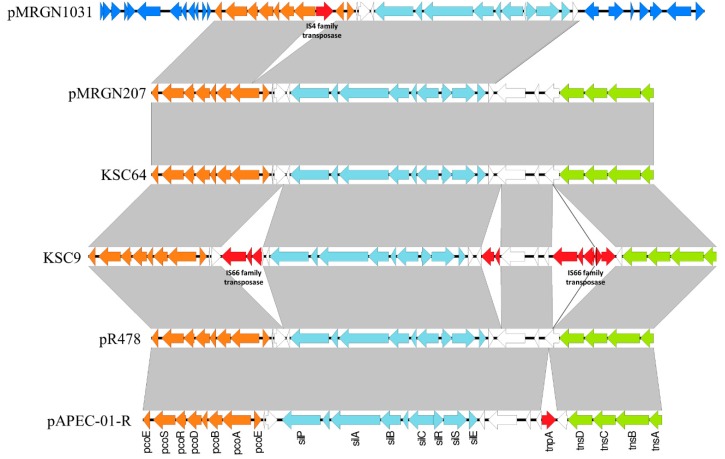
Genetic context of the *pco*–*sil*–*tns* area of four isolates from this study sequenced by PacBio. KSC1031 (plasmid pMRGN1031; GenBank accession number CP019561), KSC207 (plasmid pMRGN207; CP019559), KSC64 (CP018840), and KSC9 (CP018323) were compared to the pR478 (BXX664015) and pAPEC-O1-R (DQ517526) previously published sequences. Colors indicate the *pco* operon (orange), the *sil* operon (light blue), transposases (red), Tn*7* genes (green), and others (dark blue).

Pacific Biosciences long-read sequencing and assembly showed the *pco* gene cluster to be both plasmid and chromosomally encoded (Figure 3). The gene cluster was accompanied by the *sil* gene cluster in all four *pco*-positive isolates, and was flanked by a Tn*7*-like transposable element in three of them. Using this *pco*–*sil*–*tns* sequence as a template, short-read Illumina sequences were successfully mapped onto all but one *pco*-positive isolate (KSC1031) for the complete structure, demonstrating the highly conserved nature of this transposable element across multiple STs and plasmid types found in this study.

Both of the two chromosomal Tn*7*-like elements were found in approximately the same position, 300 kbp downstream of the preferential *glmS* insertion point for Tn*7* [29], much farther than previously reported [30,31]. Neither plasmid harboring the *pco*/*sil* gene cluster contained any known virulence factors (using Virulence Finder v1.5 [32]). In one instance, the *pcoA* gene was interrupted by a transposase, while the *sil* gene cluster was always intact (Figure 3).

### 3.5. Conjugation of a *pco*/*sil* Plasmid into Salmonella

The *pco*/*sil* plasmid (IncHI2) from *E. coli* isolate KSC207 was successfully transferred to both *Salmonella* recipient isolates, as confirmed by PCR. Using agar dilution under anaerobic conditions, a clear difference was observed between the transconjugants carrying the *sil*/*pco* gene clusters (MIC = 24), and the recipient isolates (MIC = 4; Table 2). The IncFII plasmid from *E. coli* isolate KSC1031 could not be successfully transferred to either *Salmonella* recipient.

## 4. Discussion

The plasmid-borne nature of the *pco* gene cluster [12,13,19] and the recent demonstration of its linkage with important antimicrobial resistance genes [11,17] warrant further investigations on the potential medical and public health implications of copper use in animal feed. A previous set of experiments under controlled conditions failed to demonstrate any significant selection of *pco*-positive *E. coli* isolates [2] or increase in *pco* copy numbers [22] in feces from pigs fed copper after weaning. However, *pco*-positive isolates were more frequently associated with the tetracycline resistance gene *tet*(B) than with its *tet*(A) counterpart [2]. This suggested some possible gene linkage between *pco* genes and *tet*(B) on mobile elements or clonal expansion of strains carrying both genes. The replication of these experiments described here showed the same lack of selection of *pco*-positive *E. coli* with copper concentrations in feed (125 mM) similar to those used in the field (100–250 mM; [2]). Although this does not exclude some selection in the long term or the selection of other genes, no significant effect could be detected during a single feeding period. Since none of the 82 *pco*-negative isolates sequenced carried the *sil* operon (31 of which were from samples of animals receiving copper supplementation), it is also unlikely that the copper treatment would have selected for this latter operon alone. A *pco*–*tet*(B) positive association, as well as a negative association between *pco* and *bla*_CMY_, was observed previously by Agga et al*.* [2]; however, while the direction of association was similar here, the associations were not significant (*p* = 0.344 and 0.087, respectively). We did see much higher levels of *pco* among isolates from pigs subjected to low doses of CTC (a dosage regimen for growth promotion purposes not permitted in the US since 1 January 2017), which may suggest that indirect selection of *pco* and *pco*-positive strains could occur when using tetracyclines in swine. In the present study, the prevalence of *tet*(A) was highest in the group receiving high-dose CTC (45.0% versus 22.5%) whereas the prevalence of *tet*(B) was highest in the group receiving low-dose CTC (77.5% versus 62.5%). This finding may help explain the relationship of tetracycline uses with *pco*, though such hypotheses are largely based on the previous findings of Agga et al*.* [2].

Analysis of genomic similarities between *pco*-positive isolates through cgMLST demonstrates that the *pco* genes are distributed across a variety of clonal lineages and do not cluster in only a few clear discrete groups of closely related isolates. The most frequent *pco* single-nucleotide polymorphism (SNP group C in Figure 1A and Figure 2) is also present in several STs and unrelated clonal lineages. Both observations illustrate the active horizontal transfer of the *pco* gene cluster in *E. coli* populations. However, the associations between most of the other *pco* SNP groups and STs (Figure 2) or clonal lineages (Figure 1) suggest that both a combination of short-term or local clonal spread and broader long-term horizontal gene transfer (HGT) play a role in the distribution of this gene cluster in *E. coli* from the swine population examined. Similar to the *pco*–*sil* clusters, the *tet*(A), *tet*(B), and *bla*_CMY_ genes which have been present in Enterobacteriaceae from farm animals in North America for several decades also appeared to be distributed randomly and did not cluster clearly together with *pco* genes in a discrete number of clonal lineages (Figure 1). Overall, these observations suggest that the positive and negative statistical associations observed between *pco* and *tet*(B) or *tet*(A) and *bla*_CMY_, respectively, do not rely on the expansion and contraction of a very limited number of major clonal lineages. This differs from the *bla*_CTX-M-27_ gene which was found mainly (11/12) in closely related isolates. CTX-M β-lactamases were reported in food animals much later in North America than in other continents [33] and may have emerged in swine in the US only recently. It may, therefore, be only in the early stages of its spread through HGT in bacteria from swine and still limited to a small number of clonal lineages.

The *pco* genes were located together with the *sil* cluster on a Tn*7*-like transposon structure [17,19,34] in all but one isolate in this study (KSC1031). The high transposition frequency of Tn*7* and related elements [29] may be an important reason for the distribution of the *pco*–*sil* cluster in a wide diversity of strains illustrated in the present study. Tn*7* transposons developed refined strategies to insert preferentially on mobile plasmids [29]. It may, therefore, not appear entirely surprising that the *pco* plasmid we were able to transfer by conjugation (pMRGN207) carried the full *pco*/*sil*/Tn*7*-like element, while pMRGN1031 missing the Tn*7* part of the element was not transferable. Coincidentally, the plasmid we were able to transfer was an IncHI2 plasmid, an incompatibility group already shown by others to carry *pco* genes in different geographic locations and bacterial species [17,18,35,36]. Tn*7* transposons also developed refined strategies to insert preferentially into the same selectively neutral *attTn7* chromosomal site located in proximity of the *glmS* gene [29]. However, the locations of the two chromosomal Tn*7*-like elements associated with the *pco*–*sil* cluster in the closed genome sequences generated with PacBio long reads show that this mobile element does not always insert in the same *attTn7* site or in the proximity of *glmS*. This may warrant further investigations on the transposition mechanisms of this Tn*7*-like transposable element. Together, these findings further stress the likely important role of IncHI2 plasmids and Tn*7*-like elements in the spread of the *pco*–*sil* gene clusters.

The overall structure of the region encompassing the *pco*–*sil* clusters was highly conserved and identical to pR478 [36] in two of the four isolates we investigated in detail (one plasmid-borne and the other chromosomal). This conserved region also included the *tns* gene cluster of Tn*7* and the intervening region between the *tns* and *sil* genes. This structure was described by others on several plasmids [17,19]. Insertions were present in the *pco*–*sil* clusters for the two other isolates. In one of them (chromosomal), three insertions were present in this region, but all were within open reading frames encoding putative proteins of unknown function, and were not affecting the *pco* or the *sil* gene clusters. However, in pMRGN1031, an insertion was disrupting the *pcoA* gene. This latter insertion would be expected to inactivate the copper resistance if a phenotype were detectable [14].

In addition to the loss of the *tns* gene cluster and parts of the genes upstream of the *sil* cluster already mentioned above, SNP analysis also showed that the *pco* genes in pMRGN1031 are clearly divergent from the majority of those from the other isolates of this study. This strongly supports the hypothesis that the *pco*–*sil* gene clusters on this plasmid have a longer or different evolutionary history than those found on other plasmids, and that parts of it may possibly be decaying.

The surprising initial lack of difference in susceptibility to copper between *pco*/*sil*-positive and *pco*/*sil*-negative isolates that we obtained in broth under aerobic growth conditions triggered further investigations under a variety of other conditions. Previous publications showed that the copper resistance phenotype of *pco*-positive isolates is inducible and can be triggered by preliminary incubation in subinhibitory concentrations of copper [13,36]. Subjecting our isolates to subinhibitory concentrations of copper similar to those described in these studies did not result in any change in copper MIC, and our isolates did not show any significant change in RNA transcription of the *pcoA* and *pcoD* genes after induction. Copper susceptibility of *E. coli* and *S. enterica* was tested by others with a variety of methods, including broth [17] and agar dilutions [21,37], as well as under aerobic [17,21] and anaerobic conditions [21,37]. No differences in copper MICs were observed by these authors between *pco*/*sil*-positive and -negative isolates under aerobic conditions, neither for *E. coli*, nor for *S. enterica*. However, differences were consistently observed for *S. enterica* when agar dilutions were used under anaerobic conditions [18,21]. Therefore, we also tested our *E. coli* and a few *S. enterica* isolates by agar dilution under anaerobic conditions. As expected, an evident dichotomization of MICs was visible under these conditions for *S. enterica*, but this was not the case for *E. coli*. These data are in agreement with results from others showing that copper resistance associated with the *pco* gene cluster is host-dependent [13]. The increase in MIC observed in *S. enterica* after transfer of a *pco*/*sil* plasmid from *E. coli* clearly confirmed this hypothesis.

Overall, the results from this study strongly suggest that the *pco*/*sil* gene clusters may have only a minor effect on copper MICs in typical wild-type intestinal *E. coli*, and may not represent a major selective advantage in this bacterial species in the gut of swine fed high concentrations of copper. Some of our findings are based on a relatively limited number of isolates, and confirmation on larger numbers of isolates is needed. *Escherichia coli* may represent a reservoir of mobile copper resistance determinants of potential importance for *S. enterica*. As illustrated here with pMRGN207 and by other researchers [17,18], transferable *pco*/*sil* plasmids concomitantly carry antimicrobial resistance determinants. These antimicrobial resistance determinants may help maintain these mobile plasmids, and indirectly, the *pco*–*sil* cluster in *E. coli* populations. Antimicrobial resistance may, in turn, be maintained and selected in *S. enterica* harboring these plasmids by the supplementation of feed with copper. Further animal experiments are needed to clarify the latter points. The role of IncHI2 plasmids in this context and the exact mechanisms and dynamics of transposition of Tn*7*-like transposons associated with the *pco*–*sil* gene clusters certainly also warrant further investigations, as do the respective roles and contribution of the *pco* versus *sil* genes in the observed copper resistance in *S. enterica*.

## Figures and Tables

**Table 1:** PCR targets and primers used for detection of *pco*, *sil*, and antimicrobial resistance genes.

Target	Primer	Sequence	Amplicon	Reference
*pco*	pcoD-F	CAGGAACGGTGATTGTTGTA	700 bp	[2]
	pcoD-R	CCGTAAAATCAAAGGGCTTA		
*sil*	silA_Fw	GCAAGACCGGTAAAGCAGAG	936 bp	[21]
	silA_Rv	CCTGCCAGTACAGGAACCAT		
*tet*(A)	TetA-L	GGCGGTCTTCTTCATCATGC	502 bp	[25]
	TetA-R	CGGCAGGCAGAGCAAGTAGA		
*tet*(B)	TetBGK-F2	CGCCCAGTGCTGTTGTTGTC	173 bp	[25]
	TetBGK-R2	CGCGTTGAGAAGCTGAGGTG		
*bla*_CMY_	CMYF	GACAGCCTCTTTCTCCACA	1000 bp	[25]
	CMYR	TGGACACGAAGGCTACGTA		
*bla*_CTX-M_	CTX-M-F	ATGTGCAGYACCAGTAA	512 bp	[26]
	CTX-M-R	CCGCTGCCGGTYTTATC		

**Table 2:** Isolates used in this study for copper susceptibility testing. All reported minimum inhibitory concentration (MIC) values were determined under anaerobic conditions. MBC—minimum bactericidal concentration.

Isolate	Bacteria	Serovar	*pco*	*sil*	Location (Similar to)	Broth MIC ^1^	Broth MBC ^1^	Agar MIC ^1^
KSC9	*Escherichia coli*		+	+	chromosome (IAI1)	18	18	16
KSC64	*E. coli*		+	+	chromosome (E24377A)	18	18	16
KSC207	*E. coli*		+	+	278 kbp plasmid (pR478)	18	18	16
KSC1031	*E. coli*		+	+	149 kbp plasmid (p1540)	12	12	12
KSC27	*E. coli*		−	−		12	12	16
KSC857	*E. coli*		−	−		12	12	16
ATCC 25922	*E. coli*		−	−		12	12	16
SA10689	*Salmonella*	Senftenberg	+	+	unknown	18	18	24
SA12224	*Salmonella*	Ouakam	+	+	unknown	18	18	24
SA13423	*Salmonella*	Ouakam	+	+	unknown	18	18	24
SA82699	*Salmonella*	Kentucky	−	−		18	18	4
SA81917	*Salmonella*	Kentucky	−	−		12	18	4
SA82540	*Salmonella*	Infantis	−	−		12	18	4
SA81917-TC	*Salmonella*	Kentucky	+	+	plasmid from *E. coli* KSC207	ND ^2^	ND	24
SA82540-TC	*Salmonella*	Infantis	+	+	plasmid from *E. coli* KSC207	ND	ND	24

^1^ concentration in mM; MICs are averages of three complete biological replicates. ^2^ ND (not done).
